# Twinned l-aspartic acid

**DOI:** 10.1107/S241431462500879X

**Published:** 2025-10-31

**Authors:** Martin Lutz

**Affiliations:** a Structural Biochemistry, Bijvoet Centre for Biomolecular Research, Faculty of Science, Utrecht University, Universiteitsweg 99, 3584 CG Utrecht, The Netherlands; University of Southamption, United Kingdom

**Keywords:** twinning, hydrogen bonding, non-spherical scattering factors, raw data

## Abstract

By improving the data quality with appropriate twin handling in the intensity integration, difference-Fourier maps show indications for asymmetric double-well hydrogen bonds between the carboxylate groups of the title molecule. Refinements with spherical scattering factors (IAM) and with non-spherical scattering factors (NoSpherA2) are consistent with this observation.

## Abstract 2

The natural amino acid l-aspartic acid contains two carboxylic acid groups, and the crystal structures are characterized by strong intermolecular hydrogen bonds between these groups. Data quality and proper handling of the twinning are essential for the analysis of the protonation states.

## Graphical abstract

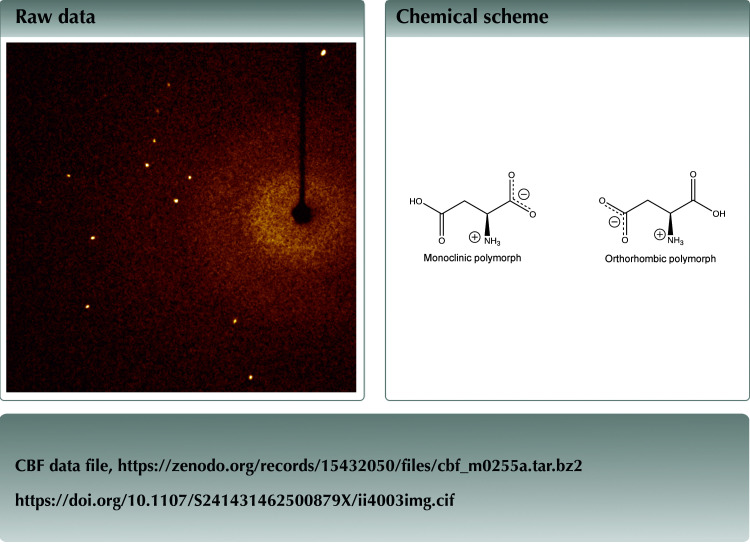

## Introduction

**Figure 1:**
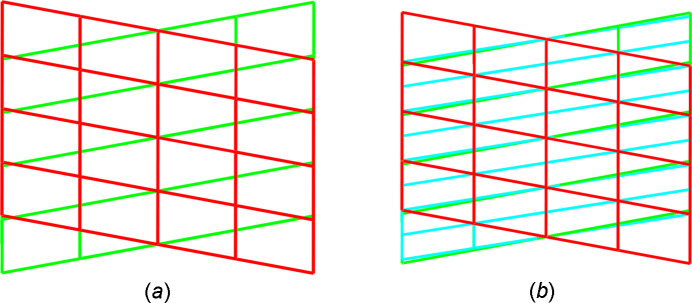
Reciprocal space geometry in the twinned title compound. (*a*) The main lattice is drawn in red, the interfering lattice in green. (*b*) Overlay of Bernal’s twin cell in cyan with the current twin interpretation in red and green.

The Cambridge Structural Database (CSD, version 5.46, November 2024; Groom *et al.*, 2016 ▸) contains seven entries for the enantiopure amino acid l-aspartic acid. Six of these entries are of the monoclinic polymorph with space group *P*2_1_, and one entry is of the orthorhombic polymorph with space group *P*2_1_2_1_2_1_ (refcode LASPRT06; Illin, 2016 ▸). In the gas phase, the most stable form of aspartic acid is a neutral molecule with a neutral NH_2_ group and two neutral carboxylic acid groups (Li *et al.*, 2007 ▸). In the monoclinic l-aspartic acid, the molecule is zwitterionic with a positively charged NH_3_ group, the main chain carboxylate is deprotonated and negatively charged, while the side-chain carboxylic acid is protonated and neutral. The orthorhombic polymorph is also zwitterionic but here the main-chain carboxylic acid is protonated and the side-chain carboxylate is deprotonated (Fig. 1 ▸).

In both the monoclinic and the orthorhombic polymorphs, the neutral carboxylic acid group is connected by an intermolecular hydrogen bond to the negatively charged carboxylate group. This raises the question whether the bridging hydrogen atom is localized on one of the two groups. Unfortunately, the data quality of the six published monoclinic structures is not sufficient to answer this question. A problem is that this monoclinic polymorph is affected by twinning. A detailed analysis of the twinning is given by (Derissen *et al.*, 1968 ▸) while the chirality of the asymmetric center seems to be wrong in their description. The present study (Table 1 ▸) aims at obtaining better data of this twinned system.

## Data processing and refinement

Indexing of the reflections with the *DIRAX* program (Duisenberg, 1992 ▸) and a high tolerance finds the twin cell, which had first been described in 1931 by Bernal ▸. In this monoclinic twin cell, the unit-cell parameters are *a* = 15.134, *b* = 6.918, *c* = 5.124 Å, β = 99.02°, and *V* = 529.81 Å^3.^ Among the *DIRAX* solutions there also is the true unit cell according to (Derissen *et al.*, 1968 ▸) as well as the second twin component. The volume of the true unit cell is half of the Bernal unit cell. The twin law is then a twofold rotation about *uvw* = [100]. The geometry of the twin lattice is shown in Fig. 1 ▸.

**Figure 2:**
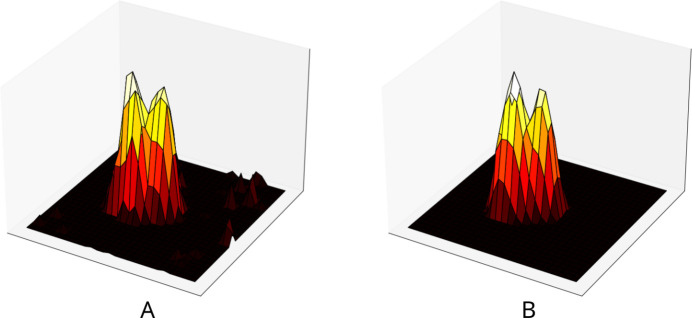
Height plot of the overlapping reflection between *hkl* = (24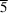) of the main lattice, and *hkl* = (2
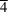
4) of the interfering lattice. (*a*) observed profile (central frame). (*b*) model profile as simulated by *Eval15*.

As consequence of the twinning, intensity integration with the *Eval15* software (Schreurs *et al.*, 2010 ▸) was based on two orientation matrices. The profile prediction involved an isotropic mosaicity of 0.275°. An example of an overlapping reflection is displayed in Fig. 2 ▸.

The result file of the *Eval15* integration contains the non-overlapping reflections of twin component 1, the non-overlapping reflections of twin component 2 and the overlapping reflections of both components. This file was read into the *TWINABS* program (Sevvana *et al.*, 2019 ▸) for absorption correction, outlier rejection, error model and merging. After manual removal of space-group absences, the merged reflection file contains 1063 non-overlapping reflections of component 1 and 494 overlapping reflections. This file was used for the structure refinement. Further details are given in Table 2 ▸.

## Data description

The true unit cell can be transformed to Bernal’s twin cell with the matrix (1, 0, 2 / 0, 1, 0 /−1, 0, 0). The determinant of this matrix is 2 and the twin index is consequently 2. On the other hand, Bernal’s primitive twin cell can be expressed as a C-centered pseudo-orthorhombic lattice with *a* = 5.124, *b* = 29.901, *c* = 6.920 Å, α = β = 90,γ = 90.74°. The twin obliquity (Le Page, 2002 ▸) of the current system is thus δ = 0.74°. The twinning fulfills Mallard’s criterion (Nespolo & Ferraris, 2005 ▸), which requires a twin index smaller than 6 and a twin obliquity smaller than 6°.

It should be noted that the twin operation needs to be a first kind operation (rotation) because a second kind operation (mirror) is not possible in this enantiopure crystal.

All hydrogen bonds are formed in the (001) plane. These hydrogen-bonded layers are connected by covalent carbon bonds into a three-dimensional network. The twin operation about vector *uvw* = [100] can be alternatively be described as a twofold rotation about vector *hkl* = (001) in the monoclinic system. Our model for the twin boundary is therefore based on the hydrogen-bonded layers. It should be noted that face (001) is also most prominent in the Bravais–Friedel–Donnay–Harker morphology prediction (BFDH; Donnay & Harker, 1937 ▸), see Table 3 ▸.

**Figure 3:**
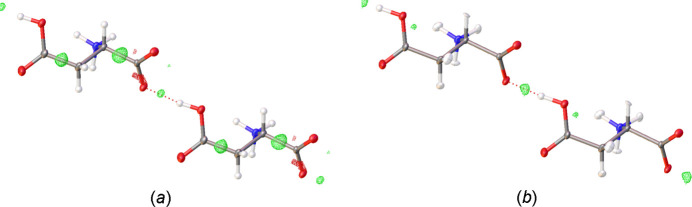
Residual electron density on the O—H⋯O hydrogen bond. (*a*) independent-atom model (contour level 0.16 e Å^−3^. (*b*) Non-spherical NoSpherA2 model (contour level 0.10 e Å^−3^.

Structure refinement with an independent-atom model in the *OLEX2* software (Dolomanov *et al.*, 2009 ▸; Bourhis *et al.*, 2015 ▸) shows a significant residual electron density on the O—H⋯O hydrogen bond (Fig. 3 ▸), which can be an indication for a double-well situation. The peak height for the modeled hydrogen atom and the residual peak are not equal. A ratio of 2:1 can be guessed. This makes it an asymmetric double-well hydrogen bond, such as is frequently observed in hydrogen bonds of moderate strength (Gilli & Gilli, 2010 ▸).

In crystal structure refinements, the use of non-spherical scattering factors can improve the reliability of hydrogen-atom positions (Woińska *et al.*, 2016 ▸). In the present case, the use of the NoSpherA2 approach (Kleemiss *et al.*, 2021 ▸) in *OLEX2* improved the *R*-values significantly. As is common with this method, the hydrogen atoms were refined with anisotropic displacement parameters. The C—H and N—H hydrogen atoms could be refined this way but the O—H hydrogen atom becomes non-positive definite. In the final refinements, the O—H hydrogen atom was therefore refined isotropically. We believe that this refinement situation confirms the double-well potential as does the remaining residual electron density on the O—H⋯O hydrogen bond (Fig. 3 ▸).

## Supplementary Material

Crystal structure: contains datablock(s) Ia, Ib, global. DOI: 10.1107/S241431462500879X/ii4003sup1.cif

Structure factors: contains datablock(s) Ia. DOI: 10.1107/S241431462500879X/ii4003Iasup2.hkl

Structure factors: contains datablock(s) Ib. DOI: 10.1107/S241431462500879X/ii4003Ibsup3.hkl

Metadata imgCIF file. DOI: 10.1107/S241431462500879X/ii4003img.cif

CheckCIF for raw data report. DOI: 10.1107/S241431462500879X/ii4003img_check.pdf

CCDC references: 2495366, 2495367

## Figures and Tables

**Table 1:** Experimental details

Raw data			
DOI	https://doi.org/10.5281/zenodo.15432050		
Data archive	Zenodo		
Data format	CBF		
Data collection			
Beamline/diffractometer			
Detector	APEXII		
Temperature (K)	150		
Radiation type	Mo 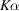		
Wavelength (Å)	0.71073		
Beam centre (mm)	30.485, 30.847		
Detector axis	−*Z*		
Detector distance (mm)	41		
Swing angle ( 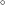 )	−26.72		
Pixel size (mm)	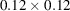		
No. of pixels	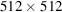		
No. of scans	7		
Exposure time per frame (s)	5		
Scan axis	Start angle, increment per frame ( 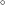 )	Scan range ( 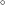 )	No. of frames
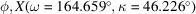	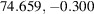	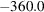	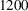
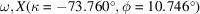	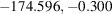	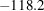	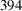
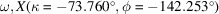	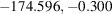	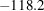	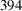
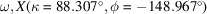	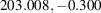	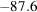	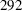
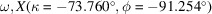	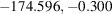	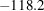	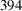
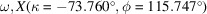	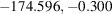	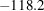	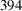
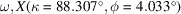	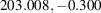	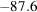	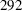

**Table 2:** Experimental details (continued)

Crystal data		
Chemical formula	C_4_H_7_NO_4_	
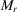	133.105	
Crystal system, space group	monoclinic, 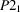	
*a*, *b*, *c* (Å)	5.1237(2), 6.9197(3), 7.6006(4)	
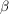 ( 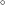 )	100.442(2)	
V (Å 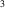 )	265.013(19)	
Z	2	
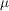 (mm 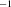 )	0.151	
Crystal size (mm)	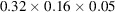	
Data processing		
Absorption correction	multi-scan (*TWINABS2012/1*; Sevvana *et al.*, 2019 ▸)	
*T*_min_, *T*_max_	0.6541, 0.7460	
Number of measured, independent and observed 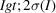 reflections	11270, 1557, 1522	
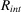	0.0272	
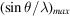 (Å 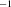 )	0.704	
Refinement		
	**Independent atom model**	**NoSpherA2**
No. of reflections	1557	1557
No. of parameters	99	141
H-atom treatment	O—H freely, C—H riding model	freely
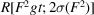 , 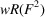 , *S*	0.0258, 0.0685, 1.0547	0.0165, 0.0369, 0.9865
Twin fraction	0.537(3)	0.5372(15)
Weighting scheme^†^	*a* = 0.0460, *b* = 0.0143	*a* = 0.0189, *b* = 0.0080
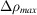 , 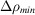 (e Å 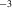 )	0.3047, −0.1757	0.3701, −00.1923
Bond precision C—C (Å)	0.0015	0.0010
		

†*a* and *b* are parameters of the *SHELXL* weighting scheme *w* = 1/[σ^2^(*F*_o_^2^) + (*a* × *P*)^2^ + *b* × *P* ] with *P* = (*F*_o_^2^ + 2*F*_c_^2^)/3.

**Table 3:** BFDH morphology prediction as calculated with the *Mercury* software (Macrae *et al.*, 2006 ▸)

Face	Perp. distance	Relative area
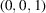	13.3784	0.153
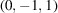	19.6933	0.081
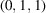	19.6933	0.081
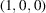	19.8458	0.086
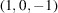	21.8311	0.034
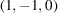	24.55	0.026
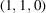	24.55	0.026
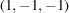	26.181	0.004
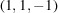	26.181	0.004
