# Supplementary material for: Twinned l-aspartic acid
Source: IUCrdata. 2025 Oct 31;10(Pt 10):x250879. doi: 10.1107/S241431462500879X (PMC12598892; doi:10.1107/S241431462500879X)
Supplement: Supplementary file 5 [file x-10-x250879-img_check.pdf]

checkImgCIF report

Powered by https://github.com/jamesrhester/ImgCIFHandler.jl

Sample image

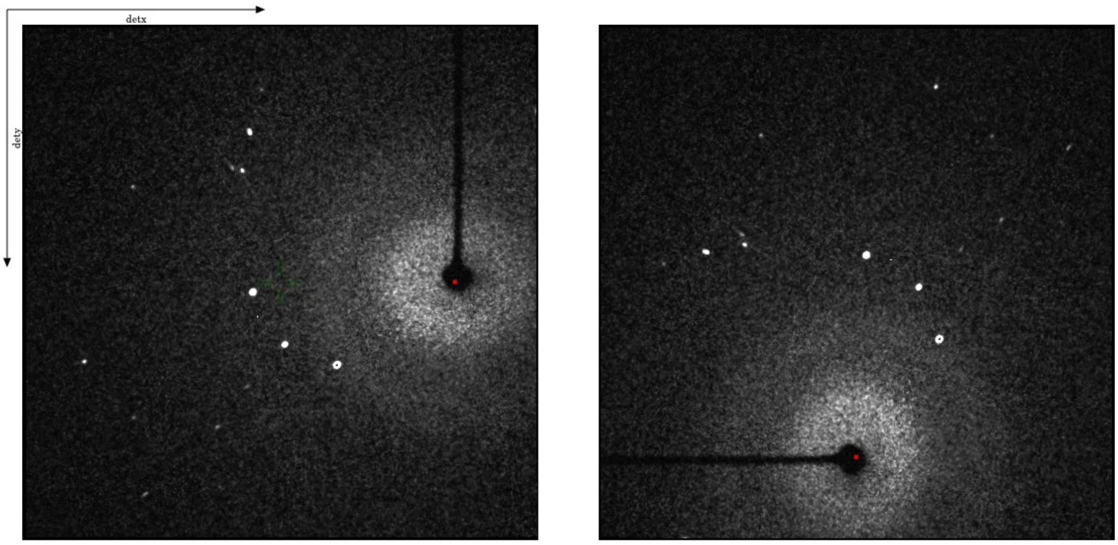

Laboratory frame

Original

```
 ImgCIF checker version 2023-03-14

Checking CIF block image

Running checks (no image download)
========================================


Testing: Required items: PASS

Testing: Data source: PASS

Testing: Axes defined: PASS

Testing: Our limitations: PASS

Testing: Detector translation: PASS

Testing: Scan range: PASS
   Range/increment match number of frames 1200.0 for scan SCAN01 (expected
1200.052002253367)
   Range/increment match number of frames 394.0 for scan SCAN02 (expected
394.01576063044496)
   Range/increment match number of frames 394.0 for scan SCAN03 (expected
394.01576063044496)
   Range/increment match number of frames 292.0 for scan SCAN04 (expected
291.98248105117)
   Range/increment match number of frames 394.0 for scan SCAN05 (expected
394.01576063044496)
   Range/increment match number of frames 394.0 for scan SCAN06 (expected
394.01576063044496)
   Range/increment match number of frames 292.0 for scan SCAN07 (expected
291.98248105117)

Testing: All frames present: PASS
```

```
   All frames present and correct for SCAN01
   All frames present and correct for SCAN02
   All frames present and correct for SCAN03
   All frames present and correct for SCAN04
   All frames present and correct for SCAN05
   All frames present and correct for SCAN06
   All frames present and correct for SCAN07

Testing: Detector surface axes used properly: PASS

Testing: Pixel size and origin described correctly: PASS

Testing: Check calculated beam centre: PASS

Testing: Check principal axis is aligned with X: PASS
Testing presence of archive:

Testing: All archives are accessible: PASS

Running checks with downloaded images
========================================

m0255a_01_0001.cbf

Testing image 1: Image type and dimensions: PASS
   WARNING: byte order provided in file containing external data pointers

Testing image 1: Overloaded values present: PASS

====End of Checks====
```
